# The Association of Knowledge and Behaviours Related to Salt with 24-h Urinary Salt Excretion in a Population from North and South India

**DOI:** 10.3390/nu9020144

**Published:** 2017-02-16

**Authors:** Claire Johnson, Sailesh Mohan, Kris Rogers, Roopa Shivashankar, Sudhir Raj Thout, Priti Gupta, Feng J. He, Graham A. MacGregor, Jacqui Webster, Anand Krishnan, Pallab K. Maulik, K. Srinath Reddy, Dorairaj Prabhakaran, Bruce Neal

**Affiliations:** 1 The George Institute for Global Health, Box M201 Missenden Rd, Sydney 2006, Australia; krogers@georgeinstitute.org (K.R.); jwebster@georgeinstitute.org.au (J.W.); bneal@georgeinstitute.org.au (B.N.); 2 School of Public Health, Department of Medicine, The University of Sydney, Sydney 2006, Australia; 3 Public Health Foundation of India, New Delhi 110070, India; smohan@phfi.org (S.M.); roopa@ccdcindia.org (R.S.); priti@ccdcindia.org (P.G.); ksrinath.reddy@phfi.org (K.S.R.); dprabhakaran@ccdcindia.org (D.P.); 4 Centre for Chronic Disease Control, New Delhi 122002, India; 5 George Institute for Global Health, Hyderabad 500034, India; traj@georgeinstitute.org.in (S.R.T.); pmaulik@georgeinstitute.org.in (P.K.M.); 6 Wolfson Institute of Preventive Medicine, Barts and The London School of Medicine & Dentistry, Queen Mary University of London, London EC1M 6BQ, UK; f.he@qmul.ac.uk (F.J.H.); g.macgregor@qmul.ac.uk (G.A.M.); 7 All India Institute of Medical Sciences, New Delhi 110029, India; kanandiyer@yahoo.com; 8 George Institute for Global Health, University of Oxford, Oxford OX1 3PA, UK; 9 Charles Perkins Centre, University of Sydney, Sydney 2050, Australia; 10 School of Public Health, Imperial College, London SW7 2AZ, UK; 11 Royal Prince Alfred Hospital, Sydney 2050, Australia

**Keywords:** India, salt, urinary sodium excretion, knowledge, attitude, behaviour

## Abstract

Consumer knowledge is understood to play a role in managing risk factors associated with cardiovascular disease and may be influenced by level of education. The association between population knowledge, behaviours and actual salt consumption was explored overall, and for more-educated compared to less-educated individuals. A cross-sectional survey was done in an age-and sex-stratified random sample of 1395 participants from urban and rural areas of North and South India. A single 24-h urine sample, participants’ physical measurements and questionnaire data were collected. The mean age of participants was 40 years, 47% were women and mean 24-h urinary salt excretion was 9.27 (8.87–9.69) g/day. Many participants reported favourable knowledge and behaviours to minimise risks related to salt. Several of these behaviours were associated with reduced salt intake—less use of salt while cooking, avoidance of snacks, namkeens, and avoidance of pickles (all *p* < 0.003). Mean salt intake was comparable in more-educated (9.21, 8.55–9.87 g/day) versus less-educated (9.34, 8.57–10.12 g/day) individuals (*p* = 0.82). There was no substantively different pattern of knowledge and behaviours between more-versus less-educated groups and no clear evidence that level of education influenced salt intake. Several consumer behaviours related to use of salt during food preparation and consumption of salty products were related to actual salt consumption and therefore appear to offer an opportunity for intervention. These would be a reasonable focus for a government-led education campaign targeting salt.

## 1. Background

Knowledge, attitudes, and behaviours can play an important role in preventing and managing risk factors associated with cardiovascular disease [1]—the leading cause of mortality globally [2,3]. In India, ischaemic heart disease and stroke are leading causes of death and hypertension is a key risk factor [4]. The prevalence of hypertension in India has increased over the past 30 years from less than 5% of adults overall to 34% of urban and 28% of rural adults today. The total numbers with hypertension are expected to almost double from 118 million in 2000 to 214 million by 2025 [5].

Eating too much salt has a clear adverse effect on blood pressure and is likely a leading cause of cardiovascular disease and stroke [6,7,8]. In 2010, 1.65 million deaths from cardiovascular causes worldwide were attributed to salt consumption of more than 5 g/day [3]. There is strong scientific evidence to show that reducing salt in the diet reduces blood pressure [9,10,11,12] and the anticipated magnitude of the vascular risk reduction that could be achieved has been defined previously: a reduction of 3 g/day over 30 years is anticipated to avert nearly 400,000 cases and about 81,000 deaths from myocardial infarction and stroke in India [13]. Additionally, the likely cost-effectiveness of national salt reduction strategies is well documented with data for India suggesting a cost of less than Rs.4400 (US$ 65) per disability-adjusted life year (DALY) averted, and great potential to prevent very large numbers of premature cardiovascular deaths [14]. All Member States of the World Health Organization (WHO), including India, have adopted a 30% reduction in mean population salt consumption by 2025 as part of the “25 by 25” initiative for the control of non-communicable diseases. This is towards the recommendation for a maximum dietary salt intake of 5 g/day for adults [15].

Over the past 20 years, National Nutrition Surveys (NNS) have shown a shift in food consumption patterns in urban and rural areas in India, largely driven by increased per capita income and changes in the food environment, making accessible a wider range of food products including highly processed products, and restaurant and fast foods [16]. Traditionally characterised by high intake of fruit, vegetables and unprocessed coarse cereals and pulses [17], the Indian diet now shows increases in average intake levels of adverse nutrients such as saturated fats, sugars and salt, now above recommended levels [8]. Concurrently, half of the population surveyed in a recent National Family Health Survey (NFHS-3) consumed less than one serving of fruit per week with individuals in the lowest socioeconomic strata consuming very low quantities, in part due to the high cost of fresh fruit and vegetables [18]. In addition, the vegetables that are consumed are often overcooked in Indian meals, leading to vital loss of micronutrients [19].

Observed differences in dietary behaviours are often attributed to socio-demographic factors such as age, sex, education and income [20,21,22,23]. Similarly, use of “discretionary” salt at the table or during food preparation is also associated with socio-economic factors, whereby individuals with lower levels of education have higher levels of discretionary salt use [24,25,26]. Knowledge, self-efficacy, attitudes and beliefs are identified reasons for diet quality variation among the different socio-economic groups [27,28,29]. Specifically, knowledge about salt has been found to be higher among older people and those with higher levels of education [30].

Health-related behavioural risk factors are widely prevalent in India but there are no related population-wide efforts for prevention targeting salt [31]. Several population surveys assessing dietary salt consumption in India estimate mean intake as >5 g/day [32,33] with results from our cross-sectional study reporting mean 24-h urinary salt excretion as 9.27 (8.87–9.69) g/day [34]. These data make a strong case for the development and implementation of a national salt reduction program; however, the identification of modifiable, mediating factors for salt intake based upon population knowledge, attitudes and behaviours would provide further insight for the development of a salt reduction strategy specific to the Indian context [1,35]. This study aimed to determine the association of knowledge, attitudes and behaviours towards salt with actual salt intake as measured by 24-h urinary sodium excretion, and to assess whether associations differed between more-educated versus less-educated individuals drawn from populations in urban and rural areas in Delhi and Haryana and Andhra Pradesh, India.

## 2. Methods

Data were collected through a cross-sectional survey in an age-stratified and sex-stratified random sample drawn from urban (slum and non-slum) and rural areas of North and South India. Ethics approval was obtained by the Human Research Ethics Committees of the Centre for Chronic Disease Control in New Delhi (approval number CCDC_IEC_10_2012) and the University of Sydney in Australia (approval number 2012/887), as well as by the Indian Health Ministry’s Screening Committee. Written informed consent was obtained from all participants. The survey was conducted between February and June 2014. The methods for participant selection and study conduct have been published elsewhere [34,36].

### 2.1. Participant Selection and Recruitment

Recruitment of participants was stratified by gender and age as well as area (urban, urban slum and rural). In North India, census enumeration blocks (CEBs) and villages were sampled at random from within the study area. Households were then selected at random and an individual from within each household was selected at random until recruitment numbers in each stratum were fulfilled. In the South, the CEBs and villages were selected to be broadly representative of those in the State using a purposive process. A census list including information about the age and sex of all inhabitants was compiled for each CEB and village and a random sample of the population was invited to participate until recruitment numbers in each stratum were filled.

### 2.2. Data Collection

Before data collection began, the local administrative body was engaged and permission to conduct the study in each area was obtained. Trained field researchers conducted interviewer-administered questionnaires over two visits within one week. Initially, consenting participants were asked questions relating to demographics, lifestyle behaviours, disease history, medication use, and knowledge, attitudes and behaviours related to salt, followed by a physical examination. Instructions to undertake a single 24-h urine collection were given. Questions about knowledge, attitudes and behaviours were adapted from the World Health Organisation/Pan American Health Organisation (WHO/PAHO) protocol for population level sodium determination [37]. The questionnaire contained 15 questions; 4 specifically on dietary habits and personal consumption; 2 relating to knowledge; and 9 assessing behaviours relevant to lowering salt intake. The participants answered on a range of different scales such as “rarely, sometimes, often”, “yes, no” and “too much, just the right amount or too little” (Table S1).

The physical examination comprised measurement of body weight (using calibrated portable Omron weight scale HN-288), and height (using calibrated Seca Brand-214 Portable Stadiometer) to the nearest 0.1 kg and 0.1 cm respectively. Weight and height were used to calculate body mass index (BMI) as weight in kilograms divided by squared height in meters. Dietary salt intake was estimated by 24-h urine collection. Urinary sodium and creatinine were determined using the ion selective electrode method for sodium analysis and the buffered kinetic Jaffe reaction without de-proteinisation for urine creatinine assay. Suspected inaccurate urine collections (i.e., urinary creatinine <4.0 mmol/day for women, or <6.0 mmol/day for men, or a 24-h urine collection of <500 mL for either sex) were excluded from the analyses. For each individual, the 24-h sodium excretion value (g/day) was calculated as the concentration of sodium in the urine (g/L) multiplied by the urinary volume (L/day).

### 2.3. Statistical Analysis

The baseline characteristics of the sample were summarised as proportions and means (95% confidence interval (CI)) overall and for subgroups defined by level of education, defined as more or less than 10 years of schooling, which was the median point in this population. The associations of knowledge, attitudes and behaviours with 24-h urinary sodium excretion were investigated by making comparisons using linear regression. Estimates were adjusted for age, sex and body mass index, which were included on the basis of their observed association with salt consumption. Subgroup analyses were done for participants with more or less than 10 years of education, which was approximately the median point among the participants surveyed. The *p* values < 0.05 were deemed significant but all findings were interpreted in light of the number of comparisons made and the broader pattern of findings across the data. Statistical analyses were conducted using SAS for Windows (version SAS 9.4).

## 3. Results

There were 1041 persons selected for the survey in Delhi and Haryana and 712 agreed to participate (68% overall response rate). The corresponding numbers for Andhra Pradesh were 1,291 and 840 (65% overall response rate). For those who agreed to take part, complete data (Knowledge, Attitudes and Behaviours (KAB) questionnaire, physical examination and a 24-h urine sample) were available for 710/712 (99%), 710/712 (99%) and 637/712 (89%) respectively in Delhi and Haryana, and 758/840 (90%), 758/840 (90%) and 758/840 (90%) respectively in Andhra Pradesh. Across both regions, there were a total of 157/1552 (10%) persons who did not return a 24-h urine sample and 438/1395 (31%) persons who returned a sample suspected to be incomplete. Participants who did not provide a complete 24-h urine collection were more likely to be older, female or from a rural site.

Accordingly, the primary analyses included 1395. Forty-seven per cent were female and 42% had more than 10 years of formal schooling. There were anticipated demographic differences between the more- and less-educated groups (Table 1). Overall salt intake was 9.27 (8.87–9.69) g/day and was not different between those with <10 years of education (9.34, 8.57–10.12 g/day) versus >10 years of education (9.21, 8.55–9.87 g/day) (*p* = 0.82).

### 3.1. Knowledge, Attitudes and Behaviours (KAB) towards Salt

The majority of participants identified the maximum salt consumption recommendation as <5 g/day (70%) and 90% identified that a diet high in salt can cause serious health problems (Table 2). About half (52%) of the participants reported that lowering salt in their diets was important but 78% of participants reported “always” adding salt to cooking. Of those who reported taking action to lower their salt intake, participants did so by using spices other than salt (98%), avoiding eating out (61%) and avoiding eating processed foods (52%).

### 3.2. Associations of Knowledge, Attitudes and Behaviours with 24-h Urinary Salt Excretion

There were four measures of knowledge and behaviour for which there were significantly higher or lower levels of urinary salt excretion (all *p* < 0.002) (Table 2) aligned with the expected effect. These related to less use of salt while cooking, avoidance of snacks and namkeens—a savoury Indian snack—and avoidance of pickles. There were no instances where a significantly higher level of urinary salt excretion was associated with a knowledge, attitude or behaviour expected to reduce intake or vice versa.

### 3.3. Associations of Knowledge, Attitudes and Behaviours with Education Level

There were several significant differences in the knowledge and behaviours reported by more- compared to less-educated individuals (all *p* < 0.017) (Table 3) and there were three instances where the levels of urinary salt excretion varied in different ways across responses to the questions for more-versus less-educated individuals (all *p* < 0.024). There was, however, no clearly discernible pattern to the variation across more- versus less-educated individuals in terms of the responses to questions and the recorded levels of salt intake (Table 4).

## 4. Discussion

There was strong evidence of an association between participant knowledge and behaviours related to salt and actual salt consumption levels as determined from assays of 24-h urine collections. This suggests that modifying population levels of these indicators of knowledge and behaviour might be an effective way of reducing population mean salt intake in India. Further, the substantial gap between the large proportion of people believing themselves to be consuming “just the right amount” of salt, and the very small proportion actually achieving the 5 g/day target, highlights the opportunity for interventions that can translate that intent into reality.

The education level of individuals may influence their acquisition of knowledge about healthy dietary practices [28,38,39] and has been associated with behaviours related to diet [29,40,41]. In this study, groups defined by different levels of education did show somewhat different results both in terms of responses to questions and corresponding levels of salt excretion, but there was no clear pattern identified whereby it was possible to ascertain particular strategies that should be targeted towards more- versus less-educated individuals. So, while any Indian intervention program targeting salt will need delivering in formats suited to individuals with a broad range of educational levels of achievement, it is not possible to specify particular messages or approaches more likely to result in a reduction in salt intake according to level of education.

Dietary patterns vary considerably around India but high levels of salt deriving from salt added during food preparation and as seasoning at the table [42] are common across many communities. In urban areas, populations are making progressively greater use of chain restaurant and fast food outlets, which often add significant quantities of salt during food preparation, whilst in rural areas salt is used in pickled fruit and vegetables dishes which are consumed in large amounts [42]. While the rapid epidemiological and nutrition transition India is undergoing [3,14] means that dietary patterns are going to evolve across the country, dietary habits will remain diverse and education programs will need to be adaptable to quite different settings.

The fairly high levels of knowledge, and the many people reporting actions to reduce salt intake are comparable to that reported in many other countries [24,30,43,44,45]. In many cases, those jurisdictions also show persisting high levels of average population salt consumption. The key difference of our findings for India compared to other countries like Australia [43] is that favourable knowledge levels and behaviours were actually associated with lower salt intake in India. This could be a chance finding or the consequence of better statistical power, but it may also be that the large proportion of dietary salt added during cooking and at the table in India makes it possible for the Indian population to control their salt intake in a way that Australians cannot. In Australia, most salt consumed is from pre-prepared packaged and restaurant foods [30]. So, while knowledgeable and motivated Indian consumers can simply leave discretionary salt out while cooking or seasoning at the table, it is much harder for Australians who would need to identify the salt content of different foods and meals and then try to find alternative lower-salt options.

This finding has important implications for any intervention to reduce salt in India. The efficacy of behavioural interventions delivered to populations has been studied mostly in high-income countries where programs typically include a combination of media and counselling activities [31]. A systematic review of such programs identified improvements in various risk factors in 22 studies, although in another 14 studies there were no benefits achieved [46]. A meta-analysis of 17 randomized controlled trials including intensive dietary behavioural interventions showed more convincing effects on dietary fat intake, serum cholesterol, urinary sodium and blood pressure [47]. On balance, the data suggest potential benefits for population-based interventions targeting salt intake [31], but data from developing country settings are few. One study undertaken in an urban community in Pakistan evaluated the effects of a household-based intervention delivered by a social worker focussing on fat and salt in a lower-middle class community. After two years, there was a reported 48% lower fat intake and 41% lower salt intake in intervention households as compared to control households [48].

There has been no dedicated population-based study of salt reduction in India, although one intervention study addressing behavioural risks for cardiovascular disease did report more consumption of fruit and vegetables and reduced intake of salt at four-year follow-up [49]. In another study done amongst at-risk Indian women, significant increases in knowledge and behaviours regarding diet-related risk factors for hypertension were observed after a community-wide education intervention that used posters, handouts, public lectures and focus groups [31]. The effectiveness of these types of programs is likely due to the fairly high intensity of engagement with community members, including one-on-one interactions and small group activities in each case. A scale-up of this type of approach would not be feasible for salt reduction in India [50] and the observed benefits cannot reasonably be generalised to population-wide settings where average exposure of individuals to the program would be much less. It is clear from experiences in Finland and the UK that population-wide salt reduction can be achieved, and community-wide education was almost certainly a key component of the success in both countries, but a novel form of intervention program tailored to India will be required [7,51].

Key strengths of this analysis are the large size of the populations included and the recruitment of individuals from regions in North and South India that span slum, urban and rural populations. This allows some capacity to generalise the findings to diverse population groups in India beyond those studied. Good participant response rates were achieved and we used weighting to control for differences in age, sex and place of residence of those sampled compared with the respective populations of the regions. However, weighting may not have fully adjusted for systematic difference in those who did and did not agree to take part. The survey also benefitted from the use of the accepted best method of quantification of dietary sodium intake based upon 24-h urine collection and the use of standardised questions about knowledge, attitudes and behaviours related to salt which are widely considered valid [43]. Multiple 24-h urine samples are required to get an accurate estimate of an individual’s usual salt intake but the associated high participant burden [52] precluded that option and we used single measurements on large numbers instead. For the estimation of average salt intake levels, this is an effective method of investigation although the between-individual variability within the population will have been over-estimated and the strengths of the associations of salt intake with the various exposures studied will probably have been under-estimated.

These data support the inclusion of population-wide education as part of a multifaceted salt reduction program for India that would likely both prevent large numbers of cases of hypertension as well as strokes and heart attacks [53,54]. Furthermore, there is a strong expectation that an intervention program could be achieved at low total cost and in a highly cost-effective way [14], making a significant contribution to the country’s efforts to deliver upon its commitment to the “25 by 25” goal of reducing chronic disease burden in the country by one quarter by 2025. The Food Safety and Standards Authority of India (FSSAI) has developed guidelines to decrease availability of foods high in fat, sugar and salt (HFSS) in and around schools through developing school canteen policies, regulating advertisements of HFSS foods to school children, including restricting celebrity endorsements and improving packaged food labelling [54]. These actions illustrate the willingness and capacity of the Government of India to act and, whilst specific to school settings, provide an example of what can be achieved with strong political leadership.

## Figures and Tables

**Table 1:** Characteristics of population overall and by level of education (*N* = 1395).

	%			
Socio-Demographic Characteristics	All %	<10 Years‘ Education	>10 Years‘ Education	*p*-Value *
Region				
Slum	6.1	7.3	4.6	0.001
Urban	29.2	19.2	40.9	
Rural	64.6	73.6	54.4	
Gender				
Male	53	46.9	60.1	0.014
Female	47	53.1	39.9	
Age Group				
20–39 years	60.4	49.7	72.9	<0.001
40–59 years	27.7	36.1	22.3	
60+ years	9.9	14.2	4.8	
Employment Status				
Employed/Domestic Duties	64.4	65.3	63.3	0.779
Unemployed/Student	35.6	34.7	36.7	
Body Mass Index (kg/m^2^)				
BMI < 25	59.7	60.9	58.3	0.653
BMI 25–30	29	29.1	28.9	
BMI 30+	11.3	10	12.9	
Blood Pressure				
SBP ≥ 140 or DBP ≥ 90	23.4	28.5	17.5	0.007
SBP < 140 and DBP < 90	76.6	71.5	82.5	
Tobacco Use				
Never	73.5	68.6	79.2	0.042
Not daily	9.6	10.2	9.0	
Daily	16.9	21.2	11.8	
Stroke				
Yes	0.4	0.5	0.3	0.435
No	99.6	99.5	99.7	
Diabetes				
Yes	6.4	8.2	4.2	0.020
No	93.6	91.8	95.8	
Chronic Kidney Disease				
Yes	2.2	1.1	3.5	0.221
No	97.8	98.9	96.5	

* *p*-value for differences in frequencies between the education groups; SBP = systolic blood pressure; DBP = diastolic blood pressure.

**Table 2:** Association between knowledge, attitudes and behaviours (KAB) and salt excretion (g/day) overall.

KAB Questions	Mean Salt (g/Day) 95% CI		
	Frequency (%)		*p*-Value *
Maximum salt consumption recommendation			
Less than 10 g (2 teaspoons or less)	19.2	9.43 (8.39, 10.47)	0.073
Less than 5 g (1 teaspoon or less)	70.0	8.93 (8.34, 9.51)	
Less than 2 g (1/2 teaspoon or less)	10.8	8.27 (7.49, 9.05)	
Does high salt intake cause health problems?			
Yes	89.6	8.66 (8.19, 9.13)	0.643
No	10.4	8.37 (7.06, 9.67)	
How much salt do you think you consume?			
Too much	8.9	9.79 (8.47, 11.10)	0.122
Just the right amount	73.3	8.67 (8.20, 9.15)	
Too little	17.8	8.18 (7.46, 8.89)	
How important to you is lowering salt in your diet?			
Very important	38.7	8.64 (8.05, 9.24)	0.928
Somewhat important	52.1	8.62 (8.00, 9.24)	
Not at all important	9.2	8.91 (7.62, 10.21)	
How often do you add salt to food at the table?			
Rarely	47.5	8.53 (7.90, 9.16)	0.627
Sometimes	20.3	8.45 (7.68, 9.21)	
Always	32.2	8.86 (8.33, 9.38)	
How often do you add salt to food when cooking?			
Rarely	15.3	7.66 (6.96, 8.35)	0.004
Sometimes	6.8	7.77 (6.55, 9.00)	
Always	77.9	8.93 (8.40, 9.45)	
Take regular action to control your salt intake?			
-check labels for sodium levels?			
Yes	3.3	8.74 (6.87, 10.62)	0.897
No	96.7	8.62 (8.14, 9.10)	
-avoid adding salt at the table?			
Yes	39.9	8.50 (7.74, 9.25)	0.571
No	60.1	8.70 (8.26, 9.15)	
-buy low-salt alternatives?			
Yes	2.9	9.37 (7.78, 10.97)	0.341
No	97.1	8.60 (8.12, 9.07)	
-avoid adding salt while cooking?			
Yes	3.6	7.10 (6.09, 8.11)	0.003
No	96.4	8.70 (8.22, 9.18)	
-use spices other than salt?			
Yes	98.1	8.67 (8.19, 9.14)	0.282
No	1.9	7.17 (4.49, 9.85)	
-avoid eating out?			
Yes	61.3	8.61 (8.10, 9.12)	0.944
No	38.7	8.64 (7.93, 9.35)	
-avoid eating snacks or namkeens?			
Yes	22.0	7.93 (7.29, 8.57)	0.006
No	78.0	8.84 (8.34, 9.35)	
-avoid eating pickles?			
Yes	18.4	7.47 (6.81, 8.13)	<0.001
No	81.6%	8.89 (8.39, 9.39)	
-avoid processed food?			
Yes	51.8	8.75 (8.06, 9.43)	0.512
No	48.2	8.49 (7.97, 9.02)	

* *p*-value for differences in mean salt intake between the responses to KAB questions.

**Table 3:** Knowledge, attitudes and behaviours by level of education (*N* = 1395).

KAB Questions	% (*n*)			
	Overall	<10 Years	>10 Years	*p*-Value *
Maximum salt consumption recommendation				
Less than 10 g (2 teaspoons or less)	19.2%	22.5%	14.1%	0.053
Less than 5 g (1 teaspoon or less)	70.0%	64.6%	76.5%	
Less than 2 g (1/2 teaspoon or less)	10.8%	12.9%	9.4%	
Does high salt intake cause health problems?				
Yes	89.6%	86.0%	94.7%	0.011
No	10.4%	14.0%	5.3%	
How much salt do you think you consume?				
Too much	8.9%	10.8%	7.4%	0.448
Just the right amount	73.3%	70.1%	75.0%	
Too little	17.8%	19.1%	17.6%	
How important to you is lowering salt in your diet?				
Very important	38.7%	45.5%	33.4%	0.058
Somewhat important	52.1%	47.8%	55.7%	
Not at all important	9.2%	6.7%	11.0%	
How often do you add salt to food at the table?				
Rarely	47.5%	49.5%	45.7%	0.680
Sometimes	20.3%	17.4%	20.1%	
Always	32.2%	33.1%	34.2%	
How often do you add salt to food when cooking?				
Rarely	15.3%	18.5%	10.4%	0.072
Sometimes	6.8%	6.8%	6.4%	
Always	77.9%	74.6%	83.2%	
Take regular action to control your salt intake?				
-check labels for sodium levels?				
Yes	3.3%	3.9%	3.4%	0.754
No	96.7%	96.1%	96.6%	
-avoid adding salt at the table?				
Yes	39.9%	37.5%	41.8%	0.326
No	60.1%	62.5%	58.2%	
- buy low-salt alternatives?				
Yes	2.9%	4.5%	1.6%	0.006
No	97.1%	95.5%	98.4%	
-avoid adding salt while cooking?				
Yes	3.6%	2.1%	4.8%	0.009
No	96.4%	97.9%	95.2%	
-use spices other than salt?				
Yes	98.1%	99.5%	96.5%	<0.001
No	1.9%	0.5%	3.5%	
-avoid eating out?				
Yes	61.3%	62.5%	56.0%	0.112
No	38.7%	37.5%	44.0%	
-avoid eating snacks or namkeens?				
Yes	22.0%	15.5%	26.2%	0.017
No	78.0%	84.5%	73.8%	
-avoid eating pickles?				
Yes	18.4%	9.6%	26.1%	<0.001
No	81.6%	90.4%	73.9%	
-avoid processed food?				
Yes	51.8%	49.2%	54.8%	0.212
No	48.2%	50.8%	45.2%	

* *p*-value for differences frequencies of responses between the educations groups.

**Table 4:** Association between KAB and salt excretion (g/day) by level of education.

KAB Questions	Mean Salt Excretion (g/Day) 95% CI		
	<10 Years	>10 Years	*p*-Value *
Maximum salt consumption recommendation			
Less than 10 g (2 teaspoons or less)	10.79 (9.26, 12.31)	9.63 (7.83, 11.42)	0.542
Less than 5 g (1 teaspoon or less)	9.72 (8.52, 10.92)	9.85 (8.96, 10.75)	
Less than 2 g (1/2 teaspoon or less)	9.21 (7.91, 10.52)	8.15 (6.83, 9.46)	
Does high salt intake cause health problems?			
Yes	9.30 (8.38, 10.23)	9.29 (8.38, 10.19)	0.082
No	10.20 (8.38, 12.01)	7.84 (5.96, 9.72)	
How much salt do you think you consume?			
Too much	11.54 (9.77, 13.30)	9.98 (8.41, 11.55)	0.082
Just the right amount	9.19 (8.27, 10.10)	9.70 (8.66, 10.73)	
Too little	9.73 (8.92, 10.54)	7.19 (6.34, 8.05)	
How important to you is lowering in your diet?			
Very important	9.93 (8.92, 10.94)	8.81 (7.79, 9.84)	0.319
Somewhat important	9.37 (8.16, 10.58)	9.50 (8.21, 10.78)	
Not at all important	9.32 (7.74, 10.90)	9.59 (7.01, 12.16)	
How often do you add salt to food at the table?			
Rarely	9.76 (8.92, 10.59)	8.94 (7.89, 9.98)	0.184
Sometimes	8.66 (7.18, 10.15)	10.54 (8.09, 13)	
Always	9.63 (8.79, 10.46)	8.85 (8.01, 9.69)	
How often do you add salt to food when cooking?			
Rarely	10.22 (9.14, 11.31)	8.23 (7.21, 9.25)	0.073
Sometimes	9.22 (6.75, 11.69)	8.10 (6.86, 9.34)	
Always	9.49 (8.57, 10.41)	9.60 (8.65, 10.55)	
Take regular action to control your salt intake?			
-check labels for sodium levels?			
Yes	7.90 (5.70, 10.10)	10.56 (9.01, 12.11)	0.017
No	9.56 (8.73, 10.39)	9.15 (8.27, 10.04)	
-avoid adding salt at the table?			
Yes	9.61 (8.65, 10.57)	8.77 (7.57, 9.96)	0.292
No	9.40 (8.49, 10.30)	9.45 (8.32, 10.59)	
-buy low-salt alternatives?			
Yes	9.35 (7.51, 11.20)	10.84 (8.60, 13.09)	0.282
No	9.50 (8.66, 10.33)	9.11 (8.21, 10.01)	
-avoid adding salt while cooking?			
Yes	8.60 (6.93, 10.27)	8.26 (6.69, 9.82)	0.943
No	9.53 (8.69, 10.36)	9.28 (8.41, 10.14)	
-use spices other than salt?			
Yes	9.51 (8.69, 10.33)	9.22 (8.37, 10.07)	0.208
No	7.09 (5.01, 9.18)	9.03 (6.20, 11.85)	
-avoid eating out?			
Yes	9.79 (8.92, 10.66)	8.57 (7.95, 9.18)	0.024
No	8.89 (7.63, 10.15)	9.89 (8.54, 11.24)	
-avoid eating snacks or namkeens?			
Yes	9.43 (8.34, 10.53)	8.51 (7.62, 9.40)	0.322
No	9.55 (8.69, 10.41)	9.50 (8.46, 10.53)	
-avoid eating pickles?			
Yes	9.10 (8.01, 10.18)	8.33 (7.47, 9.19)	0.416
No	9.56 (8.69, 10.44)	9.54 (8.52, 10.57)	
-avoid processed food?			
Yes	9.11 (8.15, 10.08)	9.96 (8.83, 11.09)	<0.001
No	9.83 (9.02, 10.64)	8.21 (7.45, 8.96)	

* *p*-value for differences in mean salt intake between the education groups.

## Supplementary Materials

The following are available online at http://www.mdpi.com/2072-6643/9/2/144/s1.
