# Supplementary material for: The Association of Knowledge and Behaviours Related to Salt with 24-h Urinary Salt Excretion in a Population from North and South India
Source: Nutrients. 2017 Feb 16;9(2):144. doi: 10.3390/nu9020144 (PMC5331575; doi:10.3390/nu9020144)
Supplement: Supplementary file 1 [file nutrients-09-00144-s001.docx]

**Supplementary Table 1. Participant knowledge, attitudes and behaviour questionnaire**

**FORM B Knowledge, Attitudes, Behaviour toward Dietary Salt**

The next questions ask about your knowledge, attitudes and behaviour towards dietary salt consumption.

Please tick the appropriate box

DIETARY HABITS

1. **Are you a vegetarian?**

⊡ Yes

⊡ No

1. **Do you eat eggs?**

⊡ Yes

⊡ No

1. **Do you eat a special diet?**

⊡ Yes

⊡ No

1. If yes, please specify: ⊡ Diabetic ⊡ High fibre ⊡ Weight reducing

⊡ Low fat ⊡ Low salt ⊡ Other …………………

1. How many years have you eaten this diet? _____/_____ (years and months)

1. **On average, how many DAYS per week would you eat meals prepared at the following:**

**Home**  **Work**

Place Number of days: └─┴─┘ Number of days: └─┴─┘

Don’t know: ⊡ Don’t know: ⊡

Other please specify ___________ Other please specify

___________

**Restaurants/take-away or home delivery Other (please specify)__________**

Number of days: └─┴─┘ Number of days: └─┴─┘

Don’t know: ⊡ Don’t know: ⊡

Other please specify ___________

1. **What type of salt do you usually use? Please check more than one if applicable:**

⊡ Common salt – Loose/crystal ⊡ Common salt - powdered

⊡ Black salt (kala namak) ⊡ Iodized salt

⊡ Sea salt ⊡ Low sodium salt

⊡ Other please specify ___________

1. **On average, how many days a week is salt added to food:**

1. **During cooking** Number of days└─┴─┘

1. **At the table**

Number of days └─┴─┘

1. **On average, how many days per week is salt added whilst kneading dough?**

Number of days: └─┴─┘ Not applicable ⊡

1. **On average, how many days per week is salt added whilst cooking rice?**

Number of days: └─┴─┘ Not applicable ⊡

Don’t know: ⊡

1. **How much salt do you think you consume?**

⊡ Too much ⊡ Just the right amount

⊡ Too little

1. **What do you think is the recommended amount of salt you should consume per day to be healthy?**

⊡ Less than 10g (2 teaspoon) ⊡ Less than 5g (1 teaspoon)

⊡ Less than 2g (1/2 teaspoon) ⊡ Other (please specify)

1. **Do you think that too much salt in your diet could cause a serious health problem?**

⊡ Yes ⊡ Don‘t know

⊡No

1. **How important to you is lowering the salt in your diet?**

⊡ Very important ⊡Somewhat important

⊡ Not at all important

1. **Do you do anything of the following on a regular basis to control your salt intake?**
2. **Look at the salt or sodium labels on food**

⊡Yes ⊡No

1. **Do not add salt at the table**

⊡Yes ⊡No

1. **Buy low salt/sodium alternatives**

⊡Yes ⊡No

1. **Do not add salt when cooking**

⊡Yes ⊡No

1. **Use spices other than salt when cooking**

⊡Yes ⊡No

1. **Avoid eating out**

⊡Yes ⊡No

1. **Avoid/minimize consumption of snacks/namkeens**

⊡Yes ⊡No

1. **Avoid/minimize consumption of pickles/papads**

⊡Yes ⊡No

1. **Avoid eating pre-made/ready-to-eat/processed foods**

⊡Yes ⊡No

1. **Other**

⊡Yes (please specify): ________________________
